# Approaching Personalized Medicine: The Use of Machine Learning to Determine Predictors of Mortality in a Population with SARS-CoV-2 Infection

**DOI:** 10.3390/biomedicines12020409

**Published:** 2024-02-09

**Authors:** Mónica Queipo, Julia Barbado, Ana María Torres, Jorge Mateo

**Affiliations:** 1 Autoimmunity and Inflammation Research Group, Río Hortega University Hospital, 47012 Valladolid, Spain; 2 Cooperative Research Network Focused on Health Results—Advanced Therapies (RICORS TERAV), 28220 Madrid, Spain; 3 Internal Medicine, Río Hortega University Hospital, 47012 Valladolid, Spain; 4 Medical Analysis Expert Group, Institute of Technology, University of Castilla-La Mancha, 16071 Cuenca, Spain; 5 Medical Analysis Expert Group, Instituto de Investigación Sanitaria de Castilla-La Mancha (IDISCAM), 45071 Toledo, Spain

**Keywords:** COVID-19, mortality, predictors, risk factors, machine learning

## Abstract

The COVID-19 pandemic demonstrated the need to develop strategies to control a new viral infection. However, the different characteristics of the health system and population of each country and hospital would require the implementation of self-systems adapted to their characteristics. The objective of this work was to determine predictors that should identify the most severe patients with COVID-19 infection. Given the poor situation of the hospitals in the first wave, the analysis of the data from that period with an accurate and fast technique can be an important contribution. In this regard, machine learning is able to objectively analyze data in hourly sets and is used in many fields. This study included 291 patients admitted to a hospital in Spain during the first three months of the pandemic. After screening seventy-one features with machine learning methods, the variables with the greatest influence on predicting mortality in this population were lymphocyte count, urea, FiO2, potassium, and serum pH. The XGB method achieved the highest accuracy, with a precision of >95%. Our study shows that the machine learning-based system can identify patterns and, thus, create a tool to help hospitals classify patients according to their severity of illness in order to optimize admission.

## 1. Introduction

Since the first cases were reported in 31 December 2019, the COVID-19 pandemic has accumulated a total of 770,875,433 confirmed cases and 6,959,316 deaths [1]. The uncontrolled spread of the virus caused by overpopulation, globalization, hyperconnectivity, and the centralization of supply chains [2] triggered a collapse of health services and resources that forced countries to take severe social actions such as isolation or lockdowns, causing serious social and economic consequences [3,4]. In Spain, health centers in the most affected areas faced problems such as inadequate intensive care capacity, insufficient equipment (both for patients and health workers), lack of medical staff, or the delay or collapse of COVID-19 helplines, which led to the cancellation of non-urgent surgeries and the need to use private health services and military facilities for public purposes [5]. In the region of Castilla y León (Spain), 585 deaths due to COVID-19 were recorded in March 2020, the first month of the pandemic in Spain, although this figure probably underestimates the actual number of deaths due to the disease during this period. In addition, the total mortality in the region increased by 775 cases in that month compared to the previous month [6].

Several studies have shown that the disease is caused by severe inflammation, a cytokine storm, and dysregulation in the levels of immune cells, especially lymphocytes. It has also been observed that some biochemical parameters involved in the disease are related to kidney and/or liver damage, such as serum urea or bilirubin levels [7]. It is also known that comorbidities related to previous diseases generally worsen the prognosis of a patient. Despite all this, the pathophysiology of the disease is not fully understood as it is a multifactorial pathology [8,9], and we do not yet understand which specific processes of the innate and adaptive immune system are decompensated or how they specifically worsen viral impact [10].

Despite the progress of scientific research, the social and hygienic policies adopted, better knowledge of the virus, the use of microbiological and serological tests such as PCR or antigen tests, and the implementation of different treatments, including the development and widespread use of vaccines, health systems still receive a significant number of infected patients with severe clinical manifestations for various reasons, including lack of vaccination, advanced age, immunosuppression, and pre-existing pathologies. On the other hand, the World Health Organization (WHO) has announced that another pandemic may occur in the near future and that the various health systems should be prepared for this event. It is therefore essential to develop techniques that allow for the rapid identification of patients at a higher risk, according to the characteristics of a given population, in order to provide a more appropriate service and improve the efficiency of health system in terms of logistics, materials, and services.

In some healthcare systems, the clinical and sociodemographic data of all patients belonging to a healthcare center are now stored in an electronic medical record system, which makes it possible to easily collect specific data on a population limited to a hospital or health area. Healthcare facilities can now choose to use these datasets to perform complex analyses using high-performance computing technologies such as machine learning (ML) methods, which are nowadays applied to an increasing number of research areas, including the healthcare field [11,12,13]. ML is defined as a branch of artificial intelligence (AI) and computer science that focuses on using data and algorithms to emulate the way in which humans learn while gradually improving their accuracy [14]. One of the advantages of this tool over other traditional statistical methods is its ability to provide accurate predictions with a high level of scalability and adaptability, finding relationships between variables using large datasets. That is why its characteristics allow ML models to be applied in areas such as diagnosis [15,16], prognosis prediction [17], drug discovery, or personalized treatments [18].

Several studies have already used this technology to answer numerous questions related to the COVID-19 pandemic, many of them aimed at identifying the factors that might be more influential in predicting a patient’s outcome at the time of admission. However, there are clear differences. Some of them agree that advanced age is a high-risk factor for mortality [19,20,21]. Banoei et al. determined that saturation level and loss of consciousness were also risk factors [22]; Kumaran et al. indicated that it was respiratory difficulties [19]; Nieto-Codesido et al. concluded that acute phase reactants had a high level of importance in poor prognoses [20]; and Izquierdo et al. reported that fever was the second most important predictor after age [23]. These studies, like others, may differ in various aspects such as the ML model used, the time interval analyzed, and/or the variables studied. What is never the same, however, are the characteristics of the population, which are completely different in each study, simply because they are carried out in different places. This shows that there are no global conclusions that can be used to establish protocols in health centers.

In the context of a new and unknown public health problem in its early stages, such as this pandemic, the main objective of this study was to demonstrate the effectiveness of the extreme gradient boost (XGB) as an ML model for studying predictors of mortality in a given population. By confirming its reliability, the method could be established as a system for evaluating risk predictors in any other given population (hospital center, health area, city, etc.), and conclusions could be drawn from data from a small number of patients (150–300) in a short time interval. This would allow for the rapid design of specific protocols for each center, based on the specific needs and characteristics of each population, making it possible to predict the care required (admission to the ward, ICU, mechanical ventilation, etc.), improve personalized patient care, and optimize resources and services.

## 2. Materials and Method

### 2.1. Data Source and Description

The source of the clinical data used in this study is the electronic medical record system of the Río Hortega University Hospital in Valladolid (Spain). Data from 291 patients hospitalized with PCR-confirmed COVID-19 infection between the 2 February 2020 and the 23 April 2020 were obtained from this platform. All the information used in this study corresponded to the first 24 h of hospitalization of these patients. Each patient was numbered with an anonymized code to preserve their privacy, and all the patients gave their informed consent. This study was conducted according to the principles of Helsinki and was approved by the Ethics Committee of the Río Hortega University Hospital.

Data were collected, retrospectively reviewed, and entered manually into a predesigned database. These data included demographics, comorbidities, chronic treatments, symptoms on admission, laboratory data, need for ICU admission, and date of death. The laboratory tests whose results were used in this study were performed at the same hospital center, and the data were entered into the aforementioned hospital electronic data storage system prior to their use in this study.

The laboratory parameters considered relevant to this study were the following: leukocytes, neutrophils, lymphocytes, monocytes, eosinophils, basophils, erythrocytes, hemoglobin, hematocrit, M.C.V., platelets, D-dimer, prothrombin activity (PT), ratio (TP), I.N.R., aPTT, aPTT ratio, derived fibrinogen, sodium, potassium, chloride, glucose, urea, creatinine, estimated glomerular filtration rate (CKD-EPI 2009), alanine aminotransferase (ALT/GPT), aspartate aminotransferase (AST/GOT), gamma glutamyl transferase (GGT), total bilirubin, alkaline phosphatase, lactate dehydrogenase (LDH), phosphate, C-reactive protein, procalcitonin, pH, FIO2, pO2/FIO2, and lactate.

### 2.2. Machine Learning Methods

In this study, the XGB method served as the reference approach. Additionally, a comparison was conducted with other ML systems. XGB is a versatile, efficient, and portable supervised learning algorithm. Its primary advantages include high execution speed, scalability, and support for parallel computing and its tendency to consistently outperform other algorithms in terms of accuracy for a wide range of data science problems [21,24,25]. For these reasons, XGB was employed in the present study to classify severe COVID-19 patients and predict variables associated with increased mortality.

In this research, various other ML algorithms were implemented to assess the performance of the proposed method. All of these algorithms are widely recognized in the scientific community. The top-performing five were selected for the comparison, including decision tree (DT) [26], Gaussian Naive Bayes (GNB) [27], Bayesian linear discriminant analysis (BLDA) [28], k-nearest neighbors (KNN) [29], and support vector machine (SVM) [30].

A brief summary of the characteristics of the implemented machine learning methods is shown below.

DTs constitute a predictive model organized in tree structures, incorporating decision rules and outcomes. The tree comprises nodes, encompassing the root, internal nodes, and leaf nodes. The depth of the tree influences the model’s generalization, and pruning techniques are employed to avoid overfitting. The construction process involves iteratively selecting features to partition the data, with the objective of maximizing homogeneity [14,26].

Gaussian Naive Bayes (GNB) is a variant that assumes a Gaussian distribution for input features. Commonly employed in classification tasks, GNB necessitates a training dataset with class-labeled examples. Parameters for the Gaussian distribution are computed for each class, and classification is performed using Bayes’ rule, which offers a probabilistic estimation [14,27].

BLDA expands upon linear discriminant analysis (LDA) by incorporating additional probabilistic assumptions. It presupposes a multivariate normal distribution within each class and applies Bayesian methodologies. BLDA proves particularly valuable when classes display distinct distributions or varying variances [14,28].

KNN is a supervised learning algorithm used for classification, relying on the majority of labels from k-nearest neighbors. It depends on a labeled training dataset, employing a selected distance metric and a specified k value. The classification process entails voting among the k neighbors to determine the label for a new point [29].

SVM is a supervised learning algorithm specifically crafted for classification purposes. It endeavors to find an optimal hyperplane in a higher-dimensional space, aiming to maximize the margin between different classes. SVM is adept at handling non-linear data through the use of the kernel trick, which involves transforming the data into a more manageable space [14,30].

**Figure 1:**
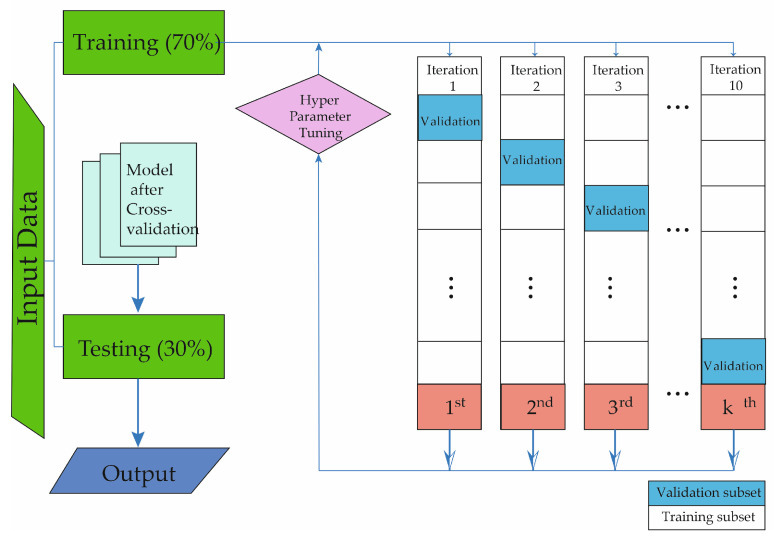
The figure shows the structure of the process for the development of the machine learning method in this study.

The models were designed using the MatLab Statistical and Machine Learning Toolbox (MatLab 2023a; The MathWorks, Natick, MA, USA). The database was divided into two segments: 70% for training and the remaining 30% for testing, with no overlap in patient data. To validate the results and prevent overfitting, 5-fold cross-validation was performed. The phases employed in this study are described in Figure 1. As depicted, the subjects of the study were initially selected. Following the implementation of the database, the ML methods were trained and validated.

Machine learning techniques typically involve one or more hyperparameters that allow for fine-tuning the algorithm during the training process. The diverse values assigned to these hyperparameters, such as the number of splits, learners, neighbors, distance metric, distant weight, kernel, box constraint level, multiclass method, etc., result in algorithms with varying prediction performances to achieve optimal results. To optimize these hyperparameters for each machine learning technique employed in this study, a Bayesian optimization approach was applied. Bayesian optimization aims to determine the hyperparameter configuration that maximizes the algorithm’s performance based on previous attempts, operating under the assumption that there is a correlation between the various hyperparameters and the algorithm’s achieved performance. The area under the AUC (receiver operating characteristic curve) and balanced accuracy served as the performance metrics to be maximized. Due to the stochastic nature of machine starting and machine learning in all simulations, 100 repetitions were conducted to calculate the mean and standard deviation values for the diverse performance metrics [14]. To mitigate the impact of data noise, ensure precise AUC calculations, and attain statistically meaningful results, the experiments were systematically replicated using a random uniform approach.

The key hyperparameters for the implemented systems are outlined below. For the SVM method, a Gaussian kernel function is utilized with the following parameters: C = 1, sigma = 0.5, numerical tolerance = 0.001, and iteration limit = 100. In the case of the DT system, adjustments are made to the base parameter estimator, with parameters set as follows: tree, maximum number of splits = 20, learning rate = 0.1, and number of learners = 40. For the GNB algorithm, the settings include usekernel: False, fL = 0, and Adjust = 0. The BLDA algorithm employs a Bayesian kernel. In the KNN method, the chosen distance metric is Euclidean, and it involves 20 neighbors. Lastly, for the XGB system, specific hyperparameters have been tuned: eta = 0.2, minimum child weight = 1, gamma = 0.3, alpha = 0.5, maximum depth = 9, lambda = 0.3, col sample by tree = 0.5, and maximum delta step = 5.

The preference for the proposed XGB over other alternative machine learning algorithms is based on its notable advantages, positioning it as a superior choice in terms of robustness, accuracy, and versatility.

Compared to SVM, XGB showcases a distinctive ability to handle intricate and high-dimensional datasets while maintaining computational efficiency. Its ensemble approach inherently introduces diversity, reducing the risk of overfitting and producing more generalized and predictive models, particularly in situations with heightened problem complexity.

In contrast to GNB, XGB excels in effectively managing irrelevant or noisy features. The integration of multiple independent decision trees allows the model to dismiss less informative variables, significantly improving its robustness and predictive efficacy.

Unlike KNN, which may be sensitive to noisy data, XGB demonstrates inherent resilience to dataset noise and variability. By constructing models based on multiple trees, the impact of outliers or errors is mitigated, ensuring greater reliability in decision making.

To sum up, the preference for XGB is substantiated by its ability to generate robust and accurate predictive models, particularly in complex environments and large datasets. Its resistance to overfitting, capability to handle irrelevant features, and versatility relative to other algorithms make it a favored choice, ensuring more dependable results and enhancing the model’s generalization capabilities.

### 2.3. Performance Evaluation

In this work, the different methods were compared with the following metrics: specificity, precision (also known as positive predictive value), recall (also known as sensitivity), balanced accuracy, degenerate Youden index (DYI), *F*_1_-score, Matthew’s correlation coefficient (MCC), Cohen’s Kappa index (CKI), receiver operating characteristic (ROC), and area under the curve (AUC) [14]. The *F*_1_ score is described as follows:

$$F_{1}score=2\frac{Precision\cdot Recall}{Precision+Recall}$$

*MCC* was also used to test the performance of the ML methods, defined as follows:

$$MCC=\frac{TP\cdot TN-FP\cdot FN}{\sqrt{\left(TP+FP\right)\left(TP+FN\right)\left(TN+FP\right)\left(TN+FN\right)}}$$

where *FP* represents the number of false positives, *TP* shows the number of true positives, *TN* is the number of true negatives, and *FN* corresponds to the number of false negatives. DYI was used to estimate the overall performance of the system [14].

## 3. Results

A cohort of 291 patients was studied. In this cohort there were 156 men and 135 women with a median age of 67 years. There were 60 patients, 35 males and 25 females, who died during hospitalization and are counted as deceased. A more detailed description is given in Table 1.

At the time of data collection, the patients had the following chronic comorbidities: diabetes mellitus (56 patients, 19%); hypertension (128 patients, 44%); dyslipidemia (104 patients, 36%); asthma (26 patients, 9%); chronic lung disease (25 patients, 9%); congestive heart failure (77 patients, 26%); overweight/obesity (28 patients, 10%); active tumors (18 patients, 6%); and other diseases (8 patients, 3%). As a result of their comorbidities, 10 patients (3%) were receiving chronic immunosuppressive treatment; 1 patient (<1%) was receiving chronic biologic treatment; and 198 patients (68%) were receiving other relevant chronic treatments.

At the time of hospitalization, the following clinical symptoms were found: cough (181 patients, 62%); sputum (35 patients, 12%); dyspnea (157 patients, 54%); anosmia (14 patients, 5%); ageusia (20 patients, 7%); nausea (22 patients, 8%); vomiting (19 patients, 7%); diarrhea (51 patients, 18%); asthenia (104 patients, 36%); dizziness (9 patients, 3%); and myalgia (39 patients, 13%). During hospitalization, 65 patients (22%) required transfer to the intensive care unit (ICU).

Data on age, temperature, and laboratory parameters at the time of admission are shown in Table 1 and Table 2.

Different ML methods were used to identify mortality risk patterns in a population with confirmed COVID-19 infection. The objective of using several algorithms was to first use the one that gave the best results in terms of predictive capacity. The results obtained using different ML methods to identify mortality risk patterns in COVID-19 patients are presented below. Table 3 and Table 4 show the performance metrics (balanced accuracy, recall, specificity, precision, MCC, *F*_1_ score, kappa, AUC, and DYI) of the ML methods used.

**Figure 2:**
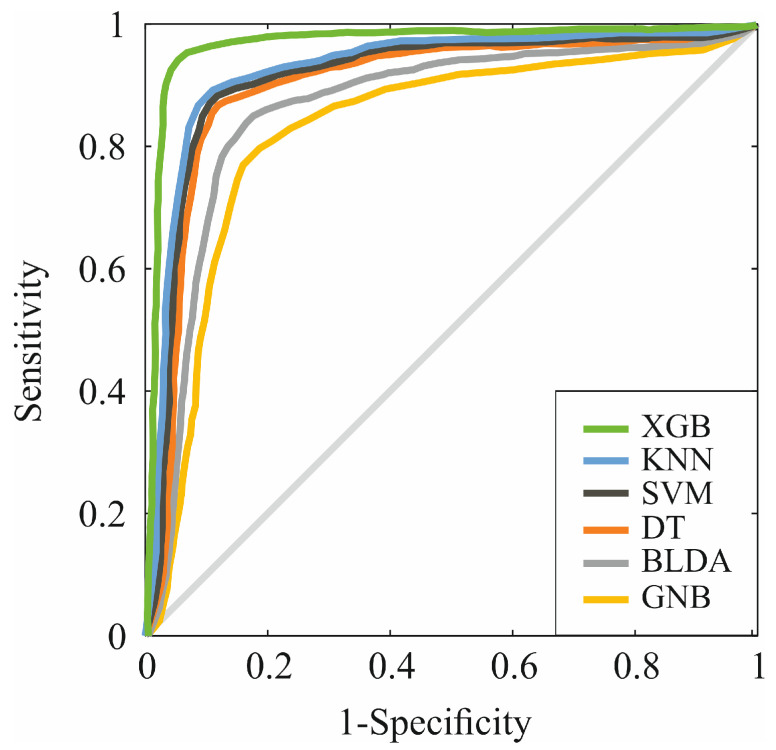
ROC curves for the six assessed machine learning predictors.

As evidenced by the data, the method proposed, XGB, is confirmed as the one with the highest acquisition and recall value. The XGB algorithm also performs consistently and uniformly with a positive prediction value greater than 95%. Additionally, the ROC curve was calculated by portraying, for each threshold value, the sensitivity and specificity measures in order to compare the classification capacity of the different ML algorithms. Figure 2 shows the results obtained. Again, the proposed XGB-based system obtains a larger area, meaning that it allows for better accuracy in prediction.

**Figure 3:**
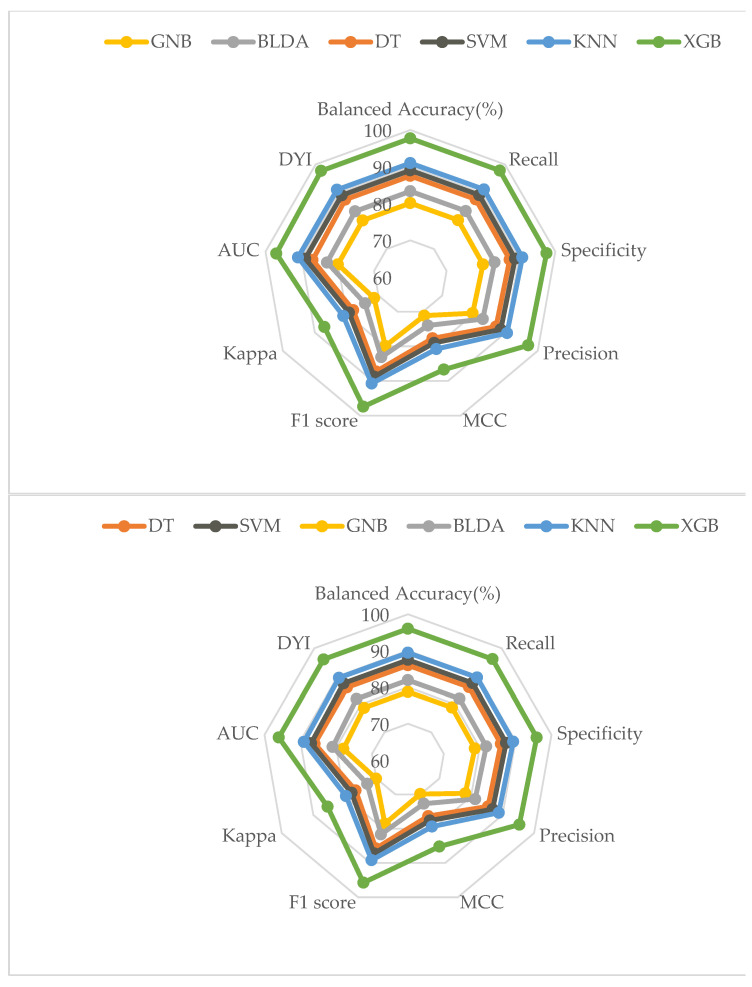
Radar plots of the variables analyzed. The **upper** one represents the training phase, and the **lower** one represents the result of the test phase.

In the present study, the model-training subsets present high scores for all the training subsets’ metrics, and, generally, they show slightly lower scores for the test subset. These similarities are due to the algorithm achieving an optimal level of training without over-fitting or under-fitting. As shown in the radar plots in Figure 3, the XGB model obtains a larger area than the other methods tested, and it is a suitable example of a well-balanced model with a high capability of generation, meaning that the algorithm gives a correct exit for each new entry.

**Figure 4:**
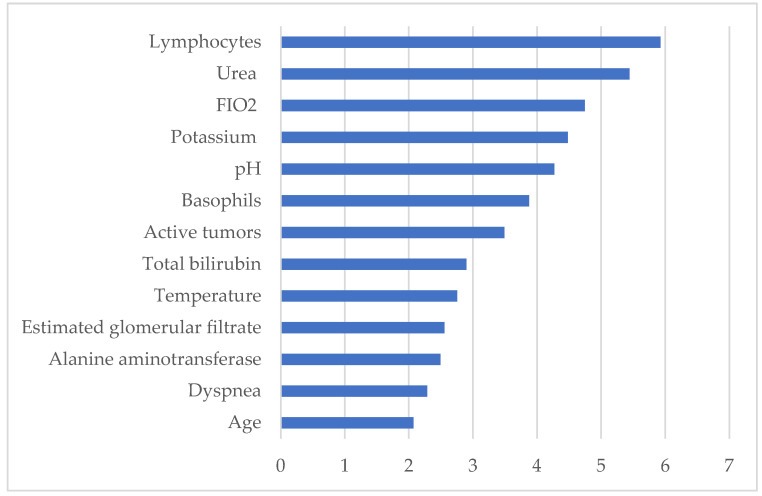
Histogram showing the 10 most relevant parameters that contribute to the mortality of COVID-19 hospitalized patients. The units in which these parameters are displayed are shown in Table 1 and Table 2.

As can be seen in Figure 4, according to the proposed XGB model, the most clinically relevant parameters contributing to the mortality of COVID-19 hospitalized patients, listed in descending order of relevance, are lymphocytes, urea, FiO2, potassium, serum pH, basophils, active tumors, total bilirubin, temperature, estimated glomerular filtrate (CKD-EPI 2009), alanine aminotransferase (ALT/GPT), dyspnea, and age. These are variables that can be easily obtained with a simple blood test at the time of hospital admission.

## 4. Discussion

Although the state of health emergency caused by the COVID-19 disease has been declared to be over [31], the virus has not disappeared, and preventive measures, both social and sanitary, are still essential to keep it at bay [32]. This possibility and the idea that another infection in the near future could reproduce a new pandemic scenario have led us to look for tools that allow the implementation of protocols as soon as possible [33] in a specific health institution. In this sense, and considering the high number of deaths caused by the COVID-19 pandemic, this study proposed a new ML model to identify clinically significant risk factors that could be measured on the first day of admission in patients hospitalized for COVID-19 during the first months of the pandemic with the aim of associating these early variables with a severe course of the disease.

The ML model based on extreme gradient boosting (XGB) was selected in our study because of its generalizability, low risk of overfitting, high interpretability [25], and high scalability [34]. XGB has been confirmed to be a reliable method for recognizing patterns in other diseases such as lupus erythematosus [16], traumatic brain injury-induced coagulopathy [35], epilepsy [36], diabetes [37], Alzheimer’s disease [38,39], HIV [40,41], or different types of cancer [42,43,44,45,46]. We, therefore, used the aforementioned ML technique to determine which factors were most predictive of disease severity in a closed group of patients hospitalized for COVID-19 during the first two months of the pandemic, a time when the population did not yet have herd immunity and had not yet been vaccinated.

The XGB model identified the patterns with the greatest weight for mortality risk in this population on the first day of hospitalization. In this context, some of them represented comorbidities such as active tumors, others clinical manifestations such as temperature and dyspnea, and, finally, analytical parameters. Among them, the first five analytical parameters were the most powerful. Serum lymphocyte count and urea level were the strongest predictors of mortality in hospitalized patients with COVID-19 in our study population. Furthermore, in a meta-analysis conducted by Tian et al. [47], which was consistent with our findings, they found that, in addition to urea and lymphocyte count, the levels of total bilirubin and ala-nine aminotransferase were also closely related to patient mortality, and they further suggested that they may indicate abnormal kidney and liver activity in patients who died. In our study, these data are reinforced by the fact that the estimated glomerular filtration rate is also among the most relevant parameters, as it has been shown in other studies to be a good predictor of admission mortality in COVID-19. In addition, our study showed a high relevance of FiO2, closely related to acute hypoxic respiratory failure-, and potassium and pH as predictors of a poor outcome and mortality, which is compatible with the results obtained by Satici et al. [48], Noori et al. [49], or Liu et al. [50]. Interestingly, hypokalemia has also been shown to be an indicator of a poorer prognosis and longer days of negative nucleic acid conversion. It is thought that this may be due to the fact that the virus interferes with ACE2, a key factor in the enzymatic cascades that maintain adequate levels of potassium in cells, causing the dysregulation of cellular physiological activity [51]. This disruption of cellular physiological systems also appears to cause the dysregulation of electrolyte homeostasis and pH imbalance [52]. Basophil count was another variable with significant weight in predicting mortality in our study. Basophils have been shown to be associated with chronic inflammatory diseases through the expression of Th17 and Th17/Th1. In the study by Murdaca et al., a significant reduction in basophils was observed during the first three days of hospitalization and returned to normal levels soon after [53].

On the other hand, in our population, active tumors were a relevant factor in relation to patient mortality, and several studies support this. Although it has been suggested that the mortality risk could be due to a suboptimal state of the immune system of these patients in relation to anticancer treatments, which would weaken the body’s response to SARS-CoV-2 [54], the findings by Lee et al. [55] and Desai et al. [56] agree that this tendency is not due to cancer-related treatments but to age, sex, and associated comorbidities. Among other clinical parameters, dyspnea was a relevant predictor in our study, consistent with few studies and in contrast to others, appearing as a parameter not closely related to mortality [57]. Finally, we found that age was a clear predictor of mortality risk, in contrast to other authors who consider that the relationship behind age being a predictor of mortality is also unclear due to the variability in the characteristics of each study [58,59].

All of the relevant parameters mentioned above are based on data that can be easily collected at the time of admission by a routine examination. Therefore, a model based on these indicators would allow for both more efficient triage and more personalized care for those patients who exhibit these risk predictors, thus improving both the care processes and the prognosis of patients infected with COVID-19.

## 5. Conclusions

The COVID-19 pandemic caused a global collapse of healthcare systems, prompting the need for new methods to identify the most at-risk patients in a timely manner. In this area, machine learning models are in the spotlight. Specifically, this study uses XGB-based modeling to identify predictors of high mortality risk in a group of patients hospitalized during the first months of the pandemic, when herd immunity was not established and vaccination had not yet begun. This analysis has allowed us to define relevant parameters that are highly useful as predictors of mortality risk at the time of hospital admission. This helps to improve patient care and treatment as well as the allocation of resources and the efficiency of health services. It has been demonstrated that the results of the proposed XGB method obtain high values of accuracy and efficiency, allowing the generation of a reliable diagnostic tool. This method could be implemented in each center, meaning that it could be used at the local level or even in each hospital to provide patients with care and attention appropriate to the demographic and environmental characteristics of each area.

## Figures and Tables

**Table 1:** Categorical baseline clinical characteristics of patients.

		Global (291 Patients)		Alive (231 Patients)		Deceased (60 Patients)	
		(*n*)	(% Total)	(*n*)	(% Total)	(*n*)	(% Total)
Sex	Men	156	54	121	42	35	11
	Women	135	46	110	38	25	9
Smoking		8	3	7	2	1	0
Drinking		2	1	1	0	1	0
Diabetes mellitus		56	19	45	15	11	4
Hypertension		128	44	97	33	31	11
Dyslipidemia		104	36	88	30	16	5
Asthma		26	9	23	8	3	1
Other chronic lung diseases		25	9	18	6	7	2
Congestive heart failure		77	26	53	18	24	8
Overweight/Obesity		28	10	24	8	4	1
Active tumors		18	6	13	4	5	2
Other relevant pathologies		8	3	8	3	0	0
Immunosuppressive chronic treatment		10	3	8	3	2	1
Biological chronic treatment		1	0	1	0	0	0
Other relevant chronic treatments		198	68	156	54	42	14
Need of ICU admission		65	22	59	20	6	2
Cough		181	62	141	48	40	14
Sputum		35	12	27	9	8	3
Dyspnea		157	54	129	44	28	10
Loss of smell (anosmia)		14	5	11	4	3	1
Loss of taste (ageusia)		20	7	17	6	3	1
Nausea		22	8	17	6	5	2
Vomiting		19	7	15	5	4	1
Diarrhea		51	18	41	14	10	3
Asthenia		104	36	87	30	17	6
Dizziness		9	3	7	2	2	1
Myalgia		39	13	29	10	10	3

**Table 2:** Numerical baseline clinical characteristics of patients.

	MEAN ± SEM		
	Global (291 Patients)	Alive (231 Patients)	Deceased (60 Patients)
Age	67.1 ± 1	64.5 ± 1.1	77.13 ± 1.5
Temperature (°C)	36.9 ± 0.1	36.9 ± 0.1	36.18 ± 0.7
Leucocytes (×10^3^ µL)	6.2 ± 0.1	6.7 ± 0.2	9.5 ± 2
Neutrophils (×10^3^ µL)	4.6 ± 0.1	5 ± 0.2	6.5 ± 0.6
Lymphocytes (×10^3^ µL)	1.02 ± 0.09	1.10 ± 0.11	0.73 ± 0.05
Monocytes (×10^3^ µL)	0.51 ± 0.02	0.53 ± 0.02	0.45 ± 0.04
Eosinophils (×10^3^ µL)	0.009 ± 0.002	0.009 ± 0.002	0.007 ± 0.004
Basophils (×10^3^ µL)	0.014 ± 0.002	0.013 ± 0.002	0.016 ± 0.005
Erythrocytes (×10^6^ µL)	4.7 ± 0	5 ± 0.2	4.6 ± 0.1
Hemoglobin (g/dL)	13.8 ± 0.1	13.8 ± 0.1	13.3 ± 0.2
Hematocrit (%)	41.5 ± 0.3	41.8 ± 0.3	40.5 ± 0.7
V.C.M. (fL)	88.3 ± 0.3	88.1 ± 0.3	88.8 ± 0.9
Platelets (×10^3^ µL)	183 ± 4.1	191.8 ± 5.5	181.9 ± 10
D-dimer (ng/mL)	459 ± 16.9	647.4 ± 69.1	1403 ± 610.6
Prothrombin activity (TP) (%)	84.7 ± 0.9	83 ± 1.2	79.3 ± 3.4
Ratio (TP)	1.1 ± 0	1.2 ± 0	1.6 ± 0.2
I.N.R.	1.1 ± 0	1.2 ± 0	1.5 ± 0.2
Patient (TTPA) (s)	31.7 ± 0.2	32.8 ± 0.5	33.9 ± 1.2
Ratio (TTPA)	1.05 ± 0.01	1.04 ± 0.01	1.09 ± 0.01
Fibrinogen (Derived) (mg/dL)	699 ± 10.2	698.1 ± 11.5	703.7 ± 22.1
Sodium (mmol/L)	135 ± 0.2	134.6 ± 0.7	134.5 ± 0.6
Potassium (mmol/L)	3.9 ± 0	5.7 ± 1.8	4 ± 0.1
Chloride (mmol/L)	99.9 ± 0.3	108.5 ± 6.5	100.4 ± 1.1
Glucose (mg/dL)	117 ± 1.4	126.8 ± 3.6	144.9 ± 6.5
Urea (mg/dL)	40.5 ± 1.1	44.3 ± 2.4	57.9 ± 3.9
Creatinine (mg/dL)	0.9 ± 0	1.7 ± 0.5	1.2 ± 0.1
Estimated glomerular filtrate (CKD-EPI 2009) (mL/min/1.73 m^2^)	66.6 ± 1.3	69.4 ± 1.5	57.7 ± 2.8
Alanine aminotransferase (ALT/GPT) (U/L)	29.7 ± 1	39.9 ± 2.2	34.9 ± 3.8
Aspartate aminotransferase (AST/GOT) (U/L)	40.3 ± 1.1	48.4 ± 2.1	60.3 ± 7.2
Gammaglutamil transferase (GGT) (U/L)	101 ± 43.5	107.5 ± 66.3	88 ± 42
Total bilirubin (mg/dL)	0.6 ± 0	0.9 ± 0.3	0.7 ± 0
Alkaline phosphatase (U/L)	70.6 ± 15.1	66.8 ± 18.8	86 ± 0
Lactate dehydrogenase (LDH) (U/L)	348 ± 7.7	347 ± 9.4	427.4 ± 29.1
Phosphate (mg/dL)	3.2 ± 0.2	3.1 ± 0.3	3.4 ± 0.4
C-reactive protein (mg/dL)	93 ± 4.5	95.9 ± 5.7	136.7 ± 14.5
Procalcitonin (ng/mL)	0.1 ± 0	0.4 ± 0.1	15.3 ± 13.1
pH	7.442 ± 0.003	7.446 ± 0.004	7.426 ± 0.008
FIO2 (%)	21 ± 0	24 ± 1	33.8 ± 4.1
pO2/FIO2	262 ± 7.5	270.8 ± 7.9	227.4 ± 19.1
Lactate (mmol/L)	1.5 ± 0	1.4 ± 0	2 ± 0.1

**Table 3:** The table shows the final results of balanced accuracy, precision, MCC, *F*_1_ score, and AUC for each machine learning method tested.

Methods	Balanced Accuracy	Precision	MCC	*F*_1_ Score	AUC
SVM	87.48 ± 0.65	86.85 ± 0.73	77.62 ± 0.54	87.22 ± 0.65	87.34 ± 0.53
DT	86.02 ± 0.54	85.40 ± 0.62	76.32 ± 0.43	85.76 ± 0.55	86.25 ± 0.47
BLDA	81.91 ± 0.79	81.33 ± 0.82	72.68 ± 0.76	81.67 ± 0.73	81.43 ± 0.76
GNB	78.75 ± 0.64	78.19 ± 0.71	69.88 ± 0.63	78.52 ± 0.66	78.35 ± 0.67
KNN	89.44 ± 0.46	88.80 ± 0.47	79.36 ± 0.43	89.17 ± 0.48	89.32 ± 0.57
XGB	96.02 ± 0.24	95.33 ± 0.26	85.20 ± 0.21	95.73 ± 0.23	96.03 ± 0.25

**Table 4:** The table presents the recall, specificity, kappa, and DYI values for each machine learning method tested.

Methods	Recall	Specificity	Kappa	DYI
SVM	87.58 ± 0.67	87.38 ± 0.74	77.88 ± 0.56	87.48 ± 0.65
DT	86.12 ± 0.56	85.91 ± 0.53	76.58 ± 0.46	86.02 ± 0.54
BLDA	82.01 ± 0.73	81.82 ± 0.75	72.92 ± 0.67	81.91 ± 0.73
GNB	78.84 ± 0.67	78.66 ± 0.69	70.11 ± 0.65	78.75 ± 0.66
KNN	89.55 ± 0.44	89.34 ± 0.45	79.63 ± 0.42	89.44 ± 0.45
XGB	96.13 ± 0.25	95.91 ± 0.23	85.48 ± 0.22	96.02 ± 0.24

## Data Availability

The datasets employed and analyzed in the current study are accessible upon reasonable request from the corresponding author. We do not have the patients’ permission to publish the data collected in this study in open access.
